# Conducting cancer research in a conflict setting: a view from the occupied Palestinian territory

**DOI:** 10.3332/ecancer.2026.2079

**Published:** 2026-02-23

**Authors:** Shaymaa AlWaheidi, Anas Ismail, Richard Sullivan, Elizabeth A Davies

**Affiliations:** 1 Centre for Cancer , Society & Public Health, King's College London, London SE1 9RT, UK; 2 Population Health Sciences Institute, Faculty of Medical Sciences, School of Geography, Politics and Sociology, Newcastle University, Newcastle upon Tyne NE2 4HH, UK; 3 Institute of Cancer Policy, Kings Health Partners Comprehensive Cancer Centre, King's College London, London SE1 9RT, UK

**Keywords:** cancer research, Palestine, ethics in conflict, Gaza

## Abstract

Cancer is now the third leading cause of death in low-income countries (65% of cancer deaths globally), accounting for over 4 million deaths every year. Despite this, research on cancer control and access to cancer care in low-income countries is limited and that in conflict settings rare. The acute and chronic nature of conflict and the vulnerability of populations caught within it, allow researchers only narrow windows of opportunity to gather and follow-up cancer data prospectively. This is usually accompanied by a lack of infrastructure and trained human resources, absence of reliable and timely data, eroded trust and poorly coordinated healthcare systems. These factors make it difficult for local researchers to conduct research, and even more difficult for international researchers to bring their skills into the setting due to movement restrictions and a lack of awareness of the local context, increasing the possibility of misjudging necessities. The problems of research in conflict go beyond the risk to the personal safety and mental health of research teams in conflict settings. Taken together, these factors could explain why opportunities for systematic data collection in conflict settings are limited despite being essential to reduce the gap in outcomes among patients with cancer living in low-income and conflict settings and those living in high-income countries. Drawing on 2 years of field research in Gaza in the occupied Palestinian territory (oPt), a conflict area in the Middle East, we present our observations from a study on breast cancer.

## Background

The occupied Palestinian territory (oPt) is a lower-middle-income country divided into two geographical territories - the West Bank, including East Jerusalem, and Gaza. Within these areas, the combined Palestinian population of about five million relies on two separate *de facto* public health systems that have been weakened by over 75 years of conflict and insecurity. The government sector Palestinian health system comes under the umbrella of the Palestinian Ministry of Health (MoH), as the main healthcare provider and financer of all public health services in the oPt. It operates in three regions: Gaza, the West Bank and East Jerusalem, with each region having its own challenges as well as resource and capability limitations. This means that referrals between regions are essential and unavoidable, but the movement restrictions imposed by Israel significantly imped effective planning and management [1].

**Figure 1.**
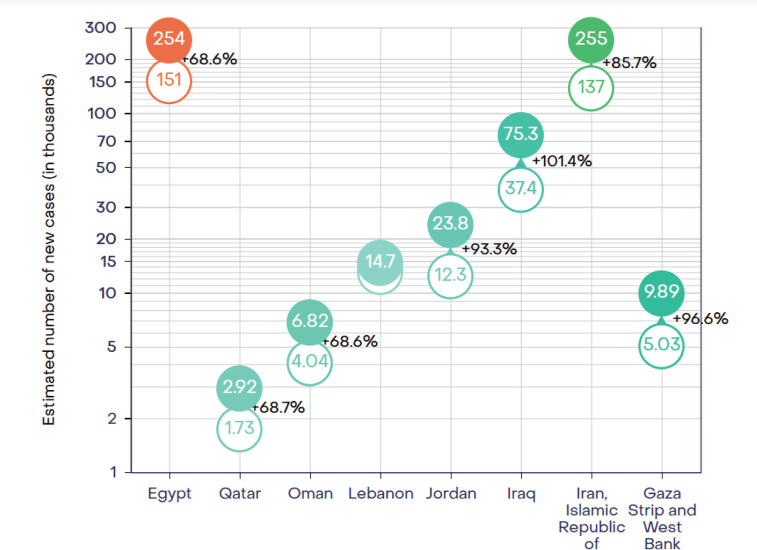
IARC Estimated number of new cases (in thousands) and projected percentage increase in incidence Eastern Mediterranean countries from 2022 to 2040 [3].

The three most common cancers in adults in the oPt are breast cancer (17%), lung cancer (11.7%) and colorectal cancer (11%) [2]. Currently, cancer causes approximately 14% of all deaths and a projection of estimates shows almost a doubling of incidence in most countries of the Eastern Mediterranean region by 2040 (Figure 1) [3].

The advanced stage at diagnosis and the pervasive shortages in basic treatment options result in a low survival and high cancer-related mortality [4]. Despite this, cancer is among the diseases with the lowest number of published research studies in the oPt [5]. The huge oncology workload, lack of financing and lack of diagnostic and treatment facilities, alongside the ongoing conflict and complex socio-political and economic crises, have had a significant negative effect not only on how the Palestinian health system operates to deliver care for cancer patients but also on efforts to support cancer research in the country. As of October 2023, Israel has destroyed most of the hospitals, including the only two dedicated cancer facilities in Gaza, which housed all paper-based cancer records, killed over 500 healthcare workers and destroyed basic infrastructure (CT scanners, X-rays, pathology labs and so on), resulting in catastrophic losses of basic chemotherapy and surgical oncology [6, 7]. The ongoing conflict has internally displaced nearly 1.7 million people in Gaza, the closure of universities and research institutions and the destruction of the Gaza cancer registry. Consequently, cancer patients have been thrust into a state of profound anxiety, cut off from their clinical support networks and unable to seek necessary treatment elsewhere due to border closures. A limited number of cancer patients have been medically evacuated to other countries, but many cancer patients have been either killed or died due to a lack of access to palliative treatment. Additionally, there is currently no capacity or capability to diagnose and treat new childhood or adult cancer cases.

Cancer research in Gaza was already challenging prior to 7th October; however, it has now become nearly impossible. This paper aims to discuss cancer research restrictions we encountered during our study to collect data on breast cancer diagnosis and treatment from paper cancer records in Gaza before and after the ongoing conflict, shedding light on the evolving landscape of cancer research in a conflict area like Gaza.

## Reflection 1: cancer data in Gaza

Most country-level data on cancer in the oPt comes from three main sources: (1) the Palestinian MoH; (2) major universities as host institutions, such as the Institute of Community and Public Health and Al-Quds University’s Faculty of Public Health; and (3) international organisations such as the World Health Organisation (WHO).

The Palestinian Cancer Registry published the first cancer report, including statistics for both the West Bank and Gaza, in 1998 [8]. From the establishment of the registry until 2008, the data of the two territories were combined in one report. The conflict, coupled with movement restrictions, scarce infrastructure and lack of trained staff, made the work of the cancer registry challenging [9, 10]. In 2007, the conflict between the two main Palestinian political parties, Fatah and Hamas, resulted in two entirely separate cancer registries, one in Gaza and the other in the West Bank. The West Bank and Gaza Cancer Registries do not publish data on site-specific incidence trends or population-based cancer survival or 5-year crude or net survival for selected cancer sites, nor do they provide data on age at diagnosis and stage distribution. Published information on grade, stage of disease and treatment also are not available. This important deficit in the literature needs to be addressed to provide clearly articulated and correct quantitative information about the quality of diagnostic care and treatment.

As a consequence, what lies behind the cancer burden figures published by the two registries is only a small amount of data collected by a small number of staff. Also, to get cancer information in Gaza from hospital notes, death certificates and referral reports, still requires manual abstraction.

A quick review of the literature is enough to obtain a clear insight into the possible explanations behind the poor quality of cancer data in conflict settings [9], but very few reports describe the lessons learned and the failures experienced in setting up and running a successful cancer registry despite ample funding from international organisations. Our experience is germane to understanding the myriad complexities of understanding cancer care in conflict.

For our research on factors affecting survival among women with breast cancer in Gaza [11], we needed to determine incident cases of female breast cancer from 1 January 2017 to 31 December 2018 and obtain detailed clinical information on these women. In an ideal world, clinical data from centralised, population-based cancer registry are generally considered the 'gold standard' for identifying incident cases of cancer. When these are not available, or information that is more recent is needed, hospital registries or other routinely collected data may be a feasible alternative source. In Gaza, these sources were not an option. First, as in many registries, the Gaza Cancer Registry collects data (mainly age, cancer type and vital status) only some time after diagnosis and treatment. Furthermore, it does not collect detailed clinical information on women with breast cancer, which is essential for epidemiological studies on survival and for assessing the appropriateness of treatment and follow-up. Second, governmental hospitals, which provide cancer care in Gaza, do not have hospital-based electronic cancer registries and they maintain all cancer records in paper format. Therefore, data collectors from the cancer registry have to scour through thousands of papers and documents by hand in the patients’ files in order to obtain the data for the registry. Understandably, the inadequate number of staff, the unorganised medical files and the paper-based nature of the archives make the job of having an up-to-date and reliable registry almost impossible. This limitation is not unique to Gaza. In many low- and middle-income countries, incomplete follow-up of patients leads to under-ascertainment of deaths and inaccurate survival estimates. Strengthening vital registration systems and improving linkage with cancer registries remain essential for generating reliable survival data.

To collect demographic and clinical data needed for our study in an ‘ethical’ way, it was important to employ ‘gatekeepers’, i.e., clinical intermediaries, to facilitate the process of data collection from paper-based cancer records. We first employed oncologists at the cancer care hospitals, but we subsequently felt that it was neither ethical nor practical for oncologists working in a fragile health system to put aside time from their extremely busy clinical practice to support our research study. We therefore found it best to balance the research team with the hospital administrative staff. There was no interruption to their work since the task required of them was part of their routine work.

Despite providing a short-term solution for the collection of cancer information in a context like Gaza, extracting and verifying hand-written clinical data was difficult and time consuming, causing unnecessary doubling of efforts among researchers. It would be costly for local researchers who do not always have external funds, as we did, and therefore impractical as a long-term solution.

Capturing incident cases of breast cancer and abstracting detailed clinical information from paper-based cancer records at extremely crowded cancer hospitals in a difficult social, political and economic context has been uniquely time-consuming, resource-intensive and ethically challenging. Flexible decision-making was therefore required to adapt to different circumstances and anticipated delays and unexpected changes. The political situation also affected fieldwork in Gaza, limiting working hours owing to intermittent internet, electricity cuts and Gaza border protests.

In order to assess the impact of cancer and the factors influencing mortality, continuous and reliable cancer registration data are essential, along with the capacity for researchers to effectively utilise this information. Since our last field visit in 2020, significant national and international efforts have been made to ensure the accuracy and currency of cancer care records, aiming to revolutionise cancer research and improve care in the country. Gaza, historically burdened by inadequate cancer registration and research infrastructure, has shown gradual progress with the establishment of new, dedicated cancer facilities. Notably, there was support for the creation of the first hospital-based cancer registry in the Gaza Cancer Centre at the Turkish-Palestinian Friendship Hospital—Gaza's first comprehensive government cancer hospital.

However, the military offensive that began in October 2023 has effectively obliterated Gaza’s emerging cancer care infrastructure. Since 2020, the Palestinian MoH, in collaboration with the first author of this paper and the WHO, had been diligently working to assess and develop the country’s first National Cancer Control Plan. This plan, published at the end of 2024 [12], was a critical milestone aimed at improving prevention, early diagnosis and treatment of the most common cancers in the oPt. The plan also sought to build capacity for governance, cancer data registration, surveillance and research, ensuring that this data could be leveraged by researchers to conduct evidence-based studies and by policymakers to enhance decision-making [12]. However, the recent destruction of key health and research infrastructure raises serious concerns about the feasibility and long-term applicability of this plan.

For Gaza’s cancer care system, a gap that was once beginning to narrow has now been dramatically widened, potentially for the foreseeable future. Even if the current war were to end tomorrow, the repercussions for cancer care would be far-reaching. In December 2023, despite the ongoing conflict, we attempted to continue our research by following up with a cohort of women to assess their 5-year survival outcomes. This endeavour, however, has proven extremely challenging due to the loss of staff and the destruction of health facilities, leaving us unsure of the whereabouts or even the existence of patient records. The health and research infrastructure in Gaza has been decimated, and rebuilding it will take decades—if not generations. According to the WHO, the damage to Gaza's healthcare system has displaced more than 50% of medical personnel and disrupted access to critical treatments [13], further exacerbating an already dire situation for cancer patients.

## Reflection 2: movement restrictions

Movement of people in and out of Gaza takes place through either the Erez border crossing between Israel and Northern Gaza or the Rafah crossing between Egypt and Southern Gaza. In addition to cancer patients, the permit system through these crossing affects healthcare providers and researchers who may also wish to travel abroad. Due to political instability, both cancer clinicians and researchers in Gaza do not receive proper training and have limited opportunities to travel abroad for postgraduate studying, conferences or training courses. The PI travelled in and out of Gaza for data collection and each time the timing of the exit was delayed and made difficult by the permit process and the frequent border closures.

In addition to the severely limited mobility across the Erez crossing, getting a permit does not help researchers, knowing that they cannot travel with their laptops. In our study, risk mitigation assessments were carried out every time the PI intended to conduct a field visit to Gaza, and extra time was provided for applying for permits. We also avoided carrying USBs or physical documents to reduce the risk of data breach of sensitive patient data, especially given how easily digital data can be accessed, copied and transferred on electronic platforms if the appropriate controls are not implemented. All data collected on women with breast cancer in Gaza were transferred onto a cloud system and were accessed through a university-secured laptop in the UK.

We had very little control over the number of permit applications the PI needed to submit before we received approval, and thus, this had a major effect on the research timeline and project funding. When submitting a request for permit approval, we would do this as early as possible, sometimes 3 months ahead of the intended travel date, in order to make sure the permit was issued on time. Still, we often only learnt whether the request was successful or not the day before the intended travel date via a text message at short notice about the decision.

Clearly, this does not allow enough planning and disrupts any timelines for research activities, overall inhibiting field visits and limiting them to the minimum.

On the other hand, travelling through the Rafah crossing is not any easier or simpler. In one of the field visits, the PI had to travel this way when her permit to travel through Erez was not approved. Travelling through Rafah requires registration at the Ministry of Interior (MoI) to join the long lists of tens of thousands of Palestinians in Gaza wanting to travel through Rafah. MoI issues weekly lists of travellers according to their travel dates. However, given that the capacity of the crossing point is only a fraction of the number of travellers, this means most travellers do not make it to the approved lists in time for their intended travel. When the PI finally made it to the list, she faced an arduous journey of nearly 24 hours to make it from Gaza to Rafah. Again, all mitigation precautions were taken by not carrying any USBs or physical documents to prevent data breaches, given the numerous physical searches travellers go through when making the subsequent journey through the insecure Sinai desert to reach Cairo.

Overall, the travelling restrictions we describe here first, limit the possibility of conducting cancer research in Gaza by anyone studying in institutions abroad. Second, when researchers do decide to conduct much-needed cancer research in Gaza, the restrictions limit their interaction with the research context, data sources and study subjects. Third, the movement restrictions extend the timelines and stretch the available funding for research, sometimes beyond capacity. Finally, the restrictions also mean little to no interaction for the local cancer researchers with researchers and research fora abroad, making them isolated and limiting both their professional development and the dissemination and influence of their research.

## Conclusion

This paper documents the profound challenges of conducting cancer research in Gaza—a region facing the collapse of its health and research infrastructure amid ongoing conflict. The destruction of cancer facilities, displacement of healthcare workers and systemic movement restrictions have not only impeded the delivery of care but also severely restricted the ability to generate and use reliable cancer data. While efforts such as the National Cancer Control Plan represented steps toward better cancer surveillance and care, the current war has reversed much of this fragile progress.

Considering the current conflict, it is difficult to predict the future of cancer care and research in Gaza. The scale and scope of destruction raise serious questions about whether systems—both clinical and academic—can be rebuilt within a reasonable timeframe without sustained international intervention. As such, there is a critical need to explore adaptable, decentralised models of cancer registration and care drawn from other conflict-affected settings (e.g., Syria, Afghanistan or Ukraine) where humanitarian actors, local researchers and governments have developed interim solutions for service delivery and data collection under duress.

To address these urgent challenges and prevent further deterioration in patient outcomes, a multipronged response is required. This includes investment in digital health records, cloud-based and remotely accessible cancer registries, and the development of ethical, secure and context-sensitive research methods. International research networks and funding bodies must play a proactive role in supporting local researchers, facilitating knowledge exchange and creating platforms that amplify voices from conflict zones.

## Conflicts of interest

None declared.

## Funding

R4HC (Research for Health in Conflict) in the MENA Region ES/P010962/1 supported the first author in building her research capacity through training courses and workshops and funding for publications on breast cancer in Gaza.

## Authors' contributions

All authors participated in the discussion and writing of the manuscript. All authors read and approved the final written version of the manuscript.

## Consent to publish declaration

Not applicable.

### Ethics and consent to participate declarations

The study was carried out in accordance with King’s College Data Protection Regulations (DPRF-17/18-7596), and prior to embarking on the research, ethical clearance was obtained from the Palestinian Health Research Committee (PHRC/HC/354/18), the Palestinian Ministry of Health (223204) and the King’s College Research Ethics Committee (HR-17/18-6982).

## Figures and Tables

**Figure 2.**
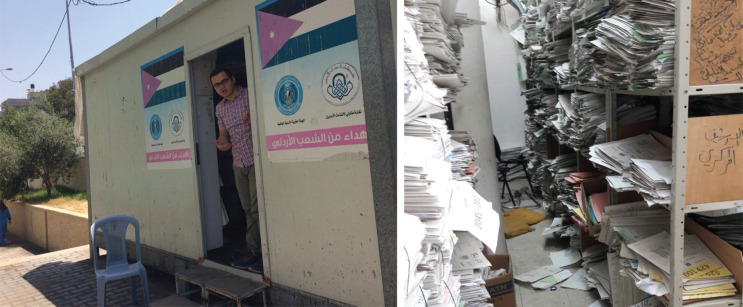
Co-author AA helped in identifying breast cancer cases diagnosed from 1 January 2017 to 31 December 2018. Cancer records were kept in a ’Portacabin’ and it was therefore massively over-crowded with not only the records of the patients but with members of staff and long queues of patients waiting for their files so that they can take them to the Oncologist who was going to see them.

**Figure 3.**
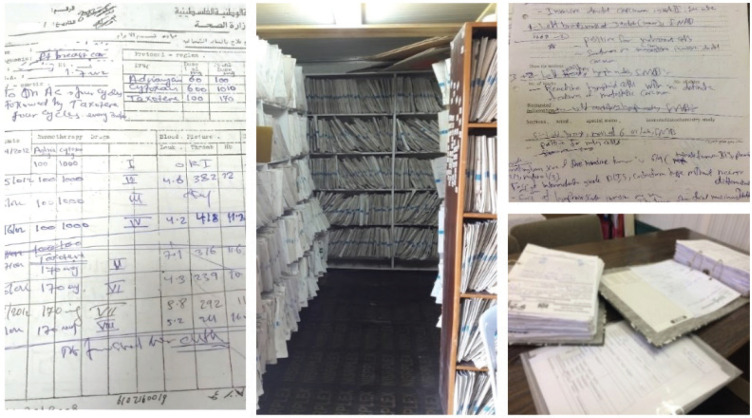
Reading biopsy reports in order to determine incident cases of breast cancer in our study and extracting treatment details for these cases
